# Process description of developing HIV prevention monitoring indicators for a province-wide pre-exposure prophylaxis (PrEP) program in British Columbia, Canada

**DOI:** 10.1371/journal.pone.0283025

**Published:** 2023-03-15

**Authors:** Lalani L. Munasinghe, Junine Toy, Katherine J. Lepik, David M. Moore, Mark Hull, Nic Bacani, Paul Sereda, Rolando Barrios, Julio S. G. Montaner, Viviane D. Lima

**Affiliations:** 1 British Columbia Centre for Excellence in HIV/AIDS, Vancouver, British Columbia, Canada; 2 St. Paul’s Hospital Ambulatory Pharmacy, St. Paul’s Hospital, Vancouver, British Columbia, Canada; 3 Division of Infectious Diseases, Department of Medicine, Faculty of Medicine, University of British Columbia, Vancouver, British Columbia, Canada; Beth Israel Deaconess Medical Center/Harvard Medical School, UNITED STATES

## Abstract

In 2018, the pre-exposure prophylaxis (PrEP) program was initiated in British Columbia (BC), Canada, providing PrEP at no cost to qualifying residents. This observational study discussed the steps to develop key evidence-based monitoring indicators and their calculation using real-time data. The indicators were conceptualized, developed, assessed and approved by the Technical Monitoring Committee of representatives from five health authority regions in BC, the BC Ministry of Health, the BC Centre for Disease Control, and the BC Centre for Excellence in HIV/AIDS. Indicator development followed the steps adopted from the United States Centers for Disease Control and Prevention framework for program evaluation in public health. The assessment involved eight selection criteria: data quality, indicator validity, existing scientific evidence, indicator informativeness, indicator computing feasibility, clients’ confidentiality maintenance capacity, indicator accuracy, and administrative considerations. Clients’ data from the provincial-wide PrEP program (January 2018—December 2020) shows the indicators’ calculation. The finalized 14 indicators included gender, age, health authority, new clients enrolled by provider type and by the health authority, new clients dispensed PrEP, clients per provider, key qualifying HIV risk factor(s), client status, PrEP usage type, PrEP quantity dispensed, syphilis and HIV testing and incident cases, and adverse drug reaction events. Cumulative clients’ data (n = 6966; 99% cis-gender males) identified an increased new client enrollment and an unexpected drop during the COVID-19 pandemic. About 80% dispensed PrEP from the Vancouver Coastal health authority. The HIV incidence risk index for men who have sex with men score ≥10 was the most common qualifying risk factor. The framework we developed integrating indicators was applied to monitor our PrEP program, which could help reduce the public health impact of HIV.

## Introduction

Despite numerous ongoing interventions to help prevent the spread of the human immunodeficiency virus (HIV), the global burden of HIV is still substantial. In 2019, an estimated 1.7 million individuals became infected worldwide [1]. In the same year, Canada recorded 2122 HIV incident cases [2], with the highest proportion of cases (39.7%) being among gay, bisexual and other men who have sex with men (gbMSM), while heterosexual contacts and people who inject drugs accounted for 28% and 22%, respectively [2]. Pre-exposure prophylaxis (PrEP), the use of daily oral antiretroviral therapy, is protective against HIV infection [3–7], as it reduces the risk of HIV acquisition among gbMSM by as much as 97% [5–8]. In 2015, the World Health Organization strongly recommended PrEP for adults with a substantial risk of HIV [4]. In response, several countries, including Canada, approved the use of tenofovir disoproxil fumarate and emtricitabine for PrEP, and many provinces subsequently initiated PrEP programs [3, 9, 10]. In January 2018, a publicly funded PrEP program was implemented in British Columbia (BC), Canada, as a supplementary initiative to the existing broad BC HIV prevention strategies such as Treatment as Prevention and harm-reduction programs [11–13]. The BC Centre for Excellence in HIV/AIDS (BC-CfE) provides PrEP free of charge to BC residents who fulfil the risk-based eligibility criteria according to its PrEP guidelines [11]. These criteria are primarily focused on gbMSM as the current HIV epidemic in BC is concentrated on this population [14], with more limited criteria for individuals who may be exposed to HIV through injection drug use or heterosexual contact. However, access to the program has been potentially limited in some cultural and ethnoracial communities due to PrEP-related stigma and unawareness [15, 16], and in remote rural areas due to topography, physical barriers, and out-of-pocket travel expenses [17, 18]. The program website provides more details: http://bccfe.ca/hiv-pre-exposure-prophylaxis-prep.

PrEP is a biomedical prevention option that requires high adherence, thus making proper routine monitoring an essential task to assess uptake, effective use and safety [19]. Unlike other HIV prevention strategies, most potential beneficiaries from PrEP are key population groups facing legal and social challenges in accessing health services [19]. Therefore, multiple factors can influence the success of PrEP program retention and adherence, including HIV-related stigma and discrimination, perceived and actual side-effect concerns, poor understanding of treatment benefits, personal choices and lifestyle [4, 19–22]. Therefore, early development and examination of robust reporting, surveillance and monitoring systems are crucial [4, 19]. In addition, timely monitoring of PrEP program outcomes helps highlight the successes and drawbacks of its performance to guide the continued optimization of the delivery of this prevention strategy. Several monitoring frameworks and indicators for examining the effective delivery of PrEP programs have been proposed [19, 23]. However, the lack of standard monitoring frameworks to assess the safe and effective delivery of PrEP, focusing on those who would benefit most, poses a challenge with indicator interpretation due to varying indicator definitions and terminologies [24]. Additionally, the inconsistent definitions and choice of indicators make evaluating PrEP programs’ performance difficult across jurisdictions [24, 25].

Over four years since the BC-CfE PrEP program implementation, actionable data have been generated to monitor the program’s progress, which is a crucial step towards its long-term success. Here, we aim to describe the steps used for the development of program monitoring indicators that can be adopted by other jurisdictions and the calculation of those indicators using live PrEP program data. We have now standardized this approach by creating a province-wide PrEP Monitoring Report (available at https://bccfe.ca/hiv-pre-exposure-prophylaxis-prep/prep-reports) that address program goals through the monitoring of key indicators to inform HIV prevention efforts across the province.

## Materials and methods

### Subjects and data sources

Data to illustrate indicators of this study were acquired from the secure and computerized BC-CfE PrEP program real-time database of clients’ data available from 1 January 2018 to 31 December 2020. The dataset was linked to a centralized province-wide population-based registry that holds data from various sources, including the BC-CfE HIV Drug Treatment Program (e.g., demographic, clinical, antiretroviral medication dispensation data) [26], the BC-CfE Pharmacovigilance Initiative (e.g., clinician-reported antiretroviral adverse drug reaction data) [27], BC Vital Statistics mortality data [28], Providence Health Care Laboratory Interface (e.g., testing data from laboratory sites, including the BC Centre for Disease Control Public Health Laboratory) [29], and the College of Physicians and Surgeons of BC (e.g., physician-related data to determine specialty type [family versus specialist physician]) [30]. Unfortunately, data were not captured for those accessing PrEP outside the program, i.e., third-party private insurers, cash-paying clients or direct online purchases [11].

### Process of developing and selecting monitoring indicators

The study adopted the Centers for Disease Control and Prevention (CDC) Framework for Program Evaluation in Public Health recommended by the United States CDC to guide the development of monitoring indicators [31]. The steps involved were: (a) engaging stakeholders, (b) describing the program, (c) focusing on the monitoring design, (d) gathering credible evidence, (e) justifying conclusions, and (f) ensuring knowledge translation and implementation. This framework was previously applied when we developed the province-wide BC-CfE Seek and Treat for Optimal Prevention HIV/AIDS Quarterly Monitoring Report Indicators [32, 33].

An interdisciplinary Technical Monitoring Committee was established, composed of representatives responsible for the healthcare delivery across BC’s five geographic health authority (HA) regions (Interior, Fraser, Vancouver Coastal, Vancouver Island, and Northern), the BC Ministry of Health, the BC Centre for Disease Control, and the BC-CfE to conceptualize, develop, review and approve the indicators. This Committee included stakeholders such as HIV specialists, epidemiologists, clinicians, data analysts, statisticians, clinical researchers, community members, and other health professionals.

Regular in-person meetings were held throughout the development process of the PrEP monitoring indicators to review and provide feedback. The Committee created a list of potential goals and objectives to develop the PrEP program monitoring indicators and described them in detail. They prioritized the objectives to identify the most relevant indicators in a feasible and timely manner. Each Committee member recognized their roles and responsibilities. The development of the monitoring indicators also focused on the availability, quality and interpretability of the PrEP program data that required monitoring.

To develop a list of indicators that define the PrEP program attributes, selected members of the Technical Monitoring Committee conducted a comprehensive literature review on existing monitoring and evaluation indicators used in HIV/AIDS surveillance, prevention and PrEP. After an extensive literature search for relevant indicators based on published material and grey literature, the whole Committee assessed the proposed indicators against eight selection criteria to minimize systematic error in decision-making, including: 1) the quality of the data source(s), 2) the validity of the indicator, 3) existing scientific evidence-based value of the indicator, 4) whether the indicator is informative, 5) feasibility of computing the indicator, 6) capacity to maintain clients’ confidentiality, 7) accuracy of estimated indicators, and 8) administrative considerations. Uniformity and consistency in making the decision were maintained using a tabulated guide provided by Lourenço et al. [32] that was slightly modified based on the consensus of the Technical Monitoring Committee (Table 1). The Committee created this list of selection criteria as informed by the CDC Framework for Program Evaluation in Public Health [31], the BC-CfE Seek and Treat for Optimal Prevention HIV/AIDS [33], the World Health Organization Implementation Tool for Pre-exposure Prophylaxis of HIV Infection [19], and the Optimizing Prevention Technology Introduction on Schedule Review and Documentation of Monitoring and Evaluation of Indicators for Oral PrEP [34].

**Table 1:** Eight-selection criteria guide for choosing indicators for decision-making.

Criteria	Recommendation
1. Data source(s) quality	An accurate and reliable source that can make data compatible with other HIV databases. Data undergo a regular assessment of quality assurance.
2. Indicator validity	Indicators have been previously validated and published in at least one peer-reviewed journal or from credible sites such as World Health Organization.
3. Scientific evidence	There is clear scientific evidence that supports the values of the indicators and meets at least some of our indicator criteria.
4. Informativeness	The indicator informs at least one of the PrEP program’s goals. Indicator outputs likely inform where meaningful change is required to improve PrEP program implementation and its effective use.
5. Computing feasibility	It is feasible to calculate the indicator using available data sources. There is the possibility of defining the indicator to provide meaningful outputs.
6. Confidentiality maintenance	The indicator does not compromise patient confidentiality.
7. Accuracy	The indicator provides a close-to-truth estimate of the outcome. The indicator can be stratified. There is sufficient and complete monitoring data to calculate the indicator. When incomplete monitoring data is available, estimated data used to calculate the indicator should not contribute to >15% of indicator calculation data.
8. Administrative consideration	The indicator was specifically requested by the BC PrEP program funders.

This table used some contents of the tabulated guide provided by Lourenço et al. [32].

All Committee members reviewed and commented on the shortlist, which consisted of the monitoring indicators that best satisfied the selection criteria or were administratively required by the BC-CfE PrEP program. Their feedback was incorporated and sent back to the Committee for re-evaluation. This process was repeated until an agreement was reached on the most relevant indicators and the best presentation method. The BC-CfE PrEP program webpage [11] provides more details on each indicator’s rationale, description, definition, calculation, use and interpretation.

### Ethics statement

The BC-CfE PrEP program is under the aegis of the BC-CfE Drug Treatment Program (DTP). The DTP is a provincially-funded clinical program mandated to i) deliver health care to individuals living with HIV and related diseases or at risk of HIV infection, ii) implement and support public health initiatives to curb HIV/AIDS, iii) monitor and evaluate these health care programs, iv) support continued quality improvement initiatives, and v) support related knowledge translation and education programs. As a result, the University of British Columbia/Providence Health Care Research Ethics Board has agreed that as a clinical registry, the DTP is able to carry out the activities mentioned above under the existing contractual agreement, known as the Shared Cost Agreement (SCA), with the BC-Ministry of Health (PharmaCare), in place since 1992 and renewed and updated regularly. Therefore, specific Research Ethics Board approval is not required for the present analysis as it falls under the mandate of the DTP. All analyses were conducted on anonymized datasets.

## Results

### PrEP monitoring indicators

A total of 14 PrEP monitoring indicators were finalized after considering the eight selection criteria shown in Table 1. Table 2 summarizes these indicators and their calculation procedure. These indicators integrate demographic, clinical characteristic risk factors of PrEP clients, health service indicators, PrEP dispensing information, and adverse drug reaction reporting. Notably, the PrEP monitoring indicators selected were client gender identity, age, regional HA, new clients enrolled by provider type and by the HA, new clients dispensed PrEP, clients per provider, key qualifying HIV risk factor(s) reported at program enrolment, client status (active or inactive), PrEP usage type, PrEP quantity dispensed, syphilis testing and incident cases, HIV testing and incident cases, and adverse drug reaction events.

**Table 2:** Finalized PrEP monitoring indicators, their definitions and their calculation procedure^a^.

Monitoring indicator	Reference	Definition	Calculation
1. Client Gender identity	[35]	The most recently recorded gender identity of unique BC-PrEP clients	Display as a count and percentage.
			**Numerator**: number of unique BC-PrEP clients dispensed PrEP, whose most recent recorded gender identity (Cisgender male, Cisgender female, Transgender male, Transgender female and Other or Unspecified) within the specified quarter.
			**Denominator:** number of unique BC-PrEP clients dispensed PrEP within the specified quarter.
2. Client Age	[3]	Age derived from the date of birth reported by the provider at the time of the PrEP prescription dispensation within the reporting quarter.	Display as a count and percentage.
			**Numerator:** number of unique BC-PrEP clients dispensed PrEP whose age corresponds to the age categories of <18, 18–28, 29–40, 41–48, and ≥49 years within the specified quarter.
			**Denominator:** total number of unique BC-PrEP clients dispensed PrEP within the specified quarter.
3. Health Authority	[11, 32]	The provider obtained and reported the clients’ HA of residence and the providers’ HA of practice.	Display as a count and percentage.
			**Numerator:** number of unique BC-PrEP clients dispensed PrEP whose HA of residence (numerator 1) and providers’ HA of practice (numerator 2) correspond to the Interior, Fraser, Vancouver Coastal, Vancouver Island, Northern, or Unknown category within the specified quarter.
			**Denominator:** total number of unique BC-PrEP clients dispensed PrEP within the specified quarter.
4. New BC-PrEP Clients Enrolled by Provider Type	[11]	New BC-PrEP clients authorized for enrolment by provider type. Physicians and nurse practitioners are differentiated based on the enrolling provider information. The physician category (family physician vs specialist physician) is identified via a data linkage with the College of Physicians and Surgeons of BC.	Display as a count and percentage.
			**Numerator:** number of new authorized BC-PrEP clients enrolled by a provider belonging to the family physician, nurse practitioner or specialist physician within the specified quarter.
			**Denominator:** total number of new BC-PrEP clients authorized for enrolment within the specified quarter.
5. New BC-PrEP Clients Enrolled by Health Authority	[11]	New BC-PrEP clients authorized for enrolment in the BC-PrEP program by HA for calendar quarters.	Display as a count and percentage.
			**Numerator:** number of new BC-PrEP clients authorized for enrolment whose HA of residence (numerator 1) and providers’ HA of practice (numerator 2), respectively, corresponds to the Interior, Fraser, Vancouver Coastal, Vancouver Island, Northern, or Unknown category within the specified quarter.
			**Denominator:** total number of new BC-PrEP clients authorized for enrolment within the specified quarter.
6. New BC-PrEP Clients Dispensed PrEP	[11]	New BC-PrEP clients authorized for enrolment who are dispensed PrEP for the first time by calendar quarters.	Display as a count and percentage.
			**Numerator:** number of new BC-PrEP clients dispensed PrEP for the first time, whose HA of residence (numerator 1) and providers’ HA of practice (numerator 2), respectively, corresponds to the Interior, Fraser, Vancouver Coastal, Vancouver Island, Northern, or Unknown category within the specified quarter.
			**Denominator:** total number of new BC-PrEP clients dispensed PrEP for the first time within the specified quarter.
7. BC-PrEP Clients per Provider	[11]	The following aspects are considered;	a. Display as a count and percentage
		a. The volume of BC-PrEP clients dispensed PrEP per distinct provider by calendar quarters.	**Numerator:** number of distinct providers whose volume of BC-PrEP program client(s) dispense PrEP corresponds to the number of clients categorized as 1, 2–5, 6–19, 20–49 and ≥50 within the specified quarter.
		b. The proportion of BC-PrEP clients covered by provider volume category.	
		The number of BC-PrEP clients dispensed PrEP is obtained by identifying the dispensed date. The distinct provider is determined based on Provider information (e.g., College ID Number and Medical Services Commission number).	**Denominator:** Total number of distinct providers within the specified quarter.
			b. Display as a percentage.
			**Numerator:** number of BC-PrEP program clients covered by each of the abovementioned provider volume categories.
			**Denominator:** total number of BC-PrEP program clients dispense PrEP within the specified quarter.
8. Key Qualifying HIV Risk Factor(s) Reported at BC-PrEP Program Enrolment	[11, 36]	Provider obtained and reported six key qualifying HIV risk factors among clients dispensed PrEP for the first time by calendar quarters. This presents in two parts below;	a. Display as a count and percentage
		a. Clients who qualified under each key risk factor at enrolment. The providers could specify more than one relevant risk factor (non-mutually exclusive categories).	**Numerator:** number of BC-PrEP clients who dispense PrEP for the first time, qualified under each key qualifying HIV risk factor at enrolment within the specified quarter.
		b. Clients belonging to different categories of qualifying key risk factor(s) specified at enrolment by BC-PrEP providers. The exact key qualifying risk factor(s) specified for each client is presented as a cumulative count updated quarterly. Thus, clients cannot belong to more than one category.	**Denominator:** total number of BC-PrEP clients dispense PrEP for the first time within the specified quarter.
		The key qualifying HIV risk factors:	b. Display as a count and percentage
		• For cis and transgender gbMSM and TGW-	**Numerator:** number of BC-PrEP clients dispense PrEP for the first time belonging to each category of key qualifying HIV risk factor(s) specified at enrolment.
		a. Infectious syphilis or rectal bacterial STI, particularly if diagnosed in the preceding 12 months.	**Denominator:** total number of BC-PrEP clients dispense PrEP for the first time at the end of the most recent available quarter.
		b. Use of nPEP on more than one occasion.	
		c. In an ongoing sexual relationship with an HIV-positive partner who is not receiving stable ART and/or does not have an HIV viral load <200 copies/mL.	
		d. HIV HIRI-MSM score ≥10.	
		• For heterosexual men and women -	
		e. Condomless vaginal or anal sex (reported at program enrolment) in conjunction with an ongoing sexual relationship with an HIV-positive partner who is not receiving stable ART and/or does not have an HIV viral load <200 copies/mL.	
		• For PWID -	
		f. Shared injection equipment (reported at program enrolment) in conjunction with having an HIV-positive injecting partner who is not receiving stable ART and/or does not have an HIV viral load <200 copies/mL.	
9. BC-PrEP Client Status (Active vs Inactive)	[11, 36]	BC-PrEP clients are considered active starting from the first PrEP prescription dispensation until the date of program discontinuation or the date the client’s prescription has lapsed >6 months (lost to follow-up).	Display as a count and percentage.
			**Numerator:** number of BC-PrEP clients corresponding to the inactive and active status in the BC-PrEP program.
			**Denominator:** total number of BC-PrEP clients within the specified quarter.
		The BC-PrEP program inactive date is the formal notification date of BC-PrEP program discontinuation. It is determined by >6 months between 2 PrEP prescription periods before the last date of Q2 2019, >6 months lapse beyond the expected PrEP refill date occurring before the last date of Q2 2019 2019, and no formal notification of program discontinuation has been received.	
10. PrEP Usage Type Among BC-PrEP Clients	[3, 11]	Daily and non-daily use of PrEP among active BC-PrEP program clients.	Display as a count and percentage.
			**Numerator:** number of active BC-PrEP clients corresponding to the PrEP usage types, i.e. prescribed daily use and prescribed non-daily use, within the specified quarter.
			**Denominator:** total number of active BC-PrEP clients within the specified quarter.
11. PrEP Quantity Dispensed (Per 30-Tablet Supply)	[11]	The prEP quantity dispensed by a pharmacy is obtained by dividing the total number of tablets ever dispensed by a unit of 30 tablets. The reporting calendar quarters are based on the date the dispensed PrEP prescription is picked up by the corresponding client if known, otherwise defaults to the date of prescription fill.	Display as a count and percentage.
			**Numerator:** PrEP quantity (per 30-tablet supply) dispensed for BC-PrEP clients whose HA of residence (numerator 1) and providers’ HA of practice (numerator 2), respectively, corresponds to the Interior, Fraser, Vancouver Coastal, Vancouver Island, Northern or Unknown category within the specified quarter
			**Denominator:** total number of 30-tablet supply units dispensed.
12. Infectious Syphilis Testing and Incident Cases	[3, 11]	Two important aspects of syphilis screening in the context of PrEP use among active BC-PrEP clients:	a. Syphilis testing surrounding a dispensed PrEP prescription
			Display as a count and percentage
			**Numerator:** among active BC-PrEP clients, the number of dispensed PrEP prescriptions in which a record of syphilis testing is present within a 30-day window prior to dispensation while allowing for a 15-day grace period following the dispensation date within the specified quarter.
		a. Whether an infectious syphilis test occurred within a 30-day window prior to a dispensed PrEP prescription while allowing for a 15-day grace period following the dispensation date.	
		b. The incident infectious syphilis cases detected among active BC-PrEP clients by calendar quarters.	**Denominator:** total number of dispensed PrEP prescriptions among active BC-PrEP clients within the specified quarter.
			b. Syphilis incident cases
		The reporting calendar quarter is based on the testing date or incident case.	Display as a count—the number of active BC-PrEP clients who had an incident syphilis case within the specified quarter.
13. HIV Testing	[3, 11, 32]	Two important aspects of HIV testing in the context of PrEP use among active BC-PrEP clients:	a. Days between the most recent negative HIV test and the date of the first dispensed PrEP prescription
			Display as a count in days (median, 25th percentile and 75th percentiles).
		a. Days between the most recent negative HIV test and the date of the first dispensed PrEP prescription.	
			Among active BC-PrEP clients, the most recent negative HIV test date is subtracted from the date of the first dispensed PrEP prescription.
		b. Whether an HIV test occurred within a 30-day window prior to a dispensed PrEP prescription while allowing for a 15-day grace period following the dispensation date.	
			b. HIV testing surrounding a dispense PrEP prescription
			Display as a count and percentage
			**Numerator:** among active BC-PrEP clients, the number of dispensed PrEP prescriptions in which a record of HIV testing is present within a 30-day window prior to dispensation while allowing for a 15-day grace period following the dispensation date within the specified quarter.
		The reporting calendar quarter is based on the date of HIV testing.	
			**Denominator:** total number of dispensed PrEP prescriptions among active BC-PrEP clients within the specified quarter.
14. Adverse Drug Reaction Events	[3, 11, 32]	ADR events among active BC-PrEP clients by calendar quarter. An ADR includes side effects or medication intolerance possibly associated with PrEP medication.	Display as a count. The number of reported ADR events among active BC-PrEP programs within the specified quarter. Counts exclude duplicate reports of the same event or ADRs classified as being unlikely to be associated with PrEP medication (based on Pharmacovigilance causality classification criteria).

^a^British Columbia Pre-exposure Prophylaxis Quarterly Monitoring Report Indicators (https://bccfe.ca/hiv-pre-exposure-prophylaxis-prep/prep-reports).

Abbreviations and acronyms: BC, British Columbia; PrEP, pre-exposure prophylaxis; HA, health authority; gbMSM, gay and bisexual men who have sex with men; TGW, transgender women; STI, Sexually Transmitted Infection; nPEP, non-occupational post-exposure prophylaxis; ART, Antiretroviral Therapy; HIRI-MSM, HIV incidence risk index for men who have sex with men; PWID, people who inject drugs; Q2, April to June calendar quarter; ADR, adverse drug reaction.

**Fig 1:**
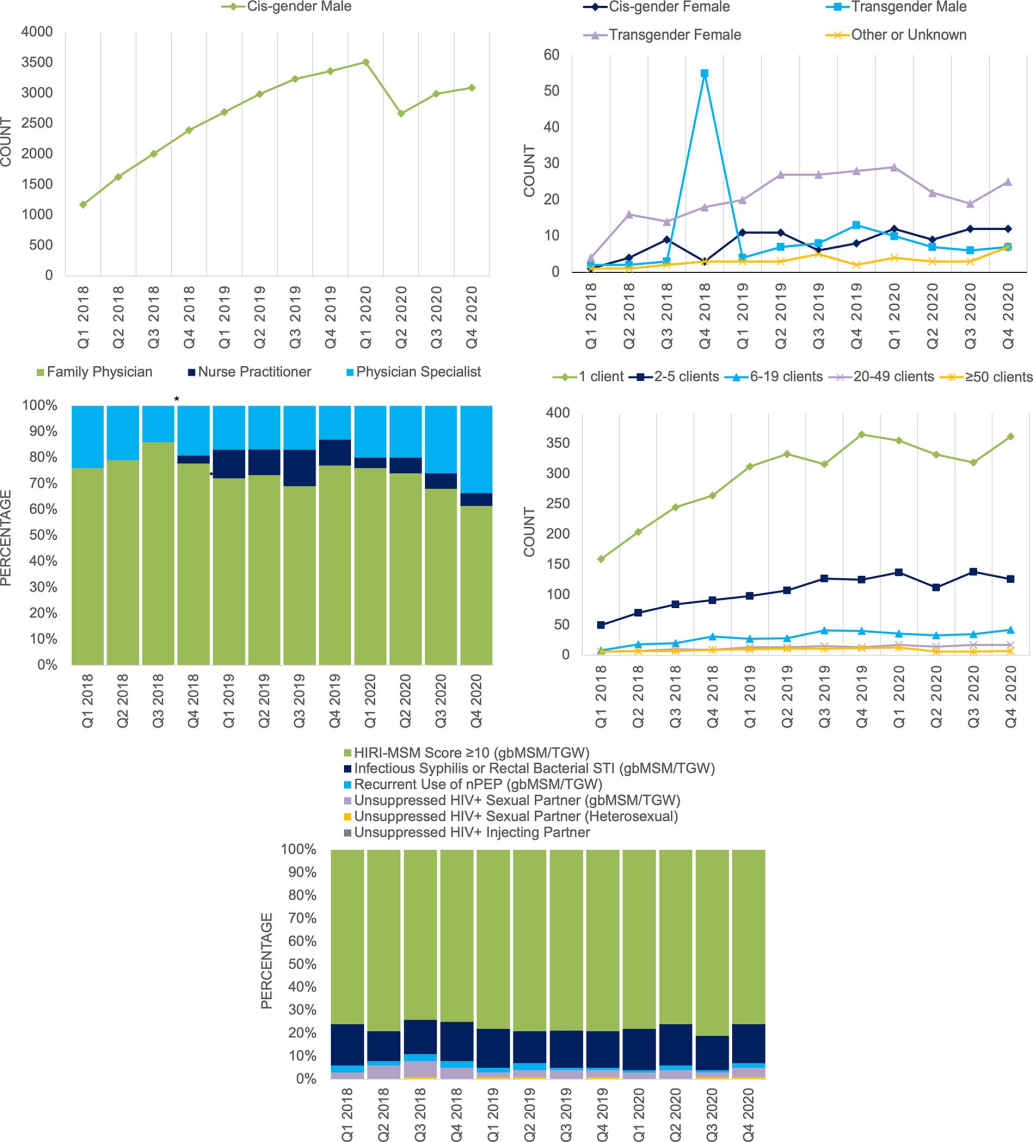
Selected monitoring indicator outputs for BC-CfE in HIV/AIDS pre-exposure prophylaxis program client database. (A) Cisgender male program clients dispensed PrEP by calendar quarters (count). (B) Program clients dispensed PrEP by gender identity except cisgender males (count). (C) New program clients enrolled by provider type (percentage). *Expansion of the nurse practitioner role to prescribe PrEP was started at the beginning of Q4 2018. (D) Providers by the number of program clients (count). (E) New program clients by non-mutually exclusive qualifying HIV risk factors reported at the enrolment (percentage).

Examples illustrating these monitoring indicators over time using live PrEP program data are depicted in Fig 1. Additionally, results obtained after calculating the indicators are explained below. We did not create a plot showing the trend of HIV incident cases to avoid the potential breaches of confidentiality in data, as very few seroconversions were identified.

Client enrollment into the program database is ongoing, and indicator values are updated as new data becomes available. Therefore, as an example, if we use program data to calculate the indicators only to the last quarter of 2020, 3139 clients received PrEP dispensation, of whom 45% were between the ages of 29 and 40 years, 98% self-identified as cisgender males, and 66% resided in our most urban HA (i.e., Vancouver Coastal). Of 315 new clients (10% of the total), 61% were enrolled by a family physician. In the same quarter, there were 554 PrEP providers, 65% of whom only had one client enrolled in the program, while 48% of clients (n = 1502) were seen by providers with ≥50 clients. There were 3387 PrEP prescriptions during this quarter, including initial prescriptions and refills. Of them, 75% had a record of syphilis testing done, and 88% had a record of HIV testing done. Fifty syphilis incident cases were identified, and two did not have any HIV testing done in that quarter. A total of 15 HIV incident cases were found from 1 January 2018 to 31 December 2020, and two of them were syphilis incident cases that were active in the program, while another two syphilis incident cases were inactive in the program.

A total of 6966 cumulative clients’ data were available in the BC-PrEP program from 1 January 2018 to 31 December 2020. In each quarter, 98–99% of the clients were identified as cisgender males, and 40–45% were aged 29–40 years. Most clients were dispensed PrEP from the Vancouver Coastal HA (67–71%) and 8–10% from Vancouver Island HA. Of 6727 clients who picked up at least one prescription, the majority (72% [n = 4829]) had only HIV Incidence Risk Index for Men who have Sex with Men [HIRI-MSM] score of ≥10 as a qualifying risk factor. The second-largest group was among those who reported having two qualifying risk factors (16% [n = 1066]): (HIRI-MSM ≥10 and previous/current infectious syphilis or rectal bacterial sexually transmitted infection). Of the 86 clients who reported having three qualifying risk factors, 51 had HIRI-MSM ≥10, prior/present infectious syphilis or rectal bacterial sexually transmitted infection, and recurrently used non-occupational post-exposure prophylaxis. Over 95% of active clients in each quarter were prescribed PrEP daily. A total of 112 adverse drug reaction events that included PrEP-related side effects or intolerance were reported, and 106 were among distinct PrEP users. The PrEP interruption was not well-defined in our cohort to identify whether these adverse drug reaction events have resulted from it; however, 84% (n = 94) of adverse drug reaction events resulted from PrEP discontinuation.

### Knowledge translation

The developed monitoring indicators can monitor regular and up-to-date information regarding the BC-PrEP program. The findings are disseminated to clinicians, medical practitioners, epidemiologists, public health personnel, funders and other stakeholders across BC to inform the best clinical and public health practice in relation to PrEP. Ultimately, the provincial HAs are responsible for designing and implementing strategies and interventions to improve PrEP delivery and outcomes for their clients.

## Discussion

We described developing an indicator framework for monitoring a province-wide PrEP program in BC, Canada. Our study summarizes several aspects of the program, including demographic, programmatic and clinical characteristics. The proposed monitoring indicators could also be stratified by geographic HA region (possibly combining the regions with low population densities) to identify people who access PrEP at the health region level, which enables prioritizing such regions for additional preventive measures. Although our data did not allow for further stratification by gender, age and clinic type due to small counts, this information would also highlight subgroups for targeted interventions to improve access and outcomes related to the BC-PrEP program across the province.

The monitoring indicators should focus on reducing HIV infections and maintaining the program’s safety and effective use [19]. Due to successful HIV management through our PrEP program, the live data we used to calculate the indicators had very few HIV seroconversions and could not create a time trend. However, it is also possible that HIV seroconversion occurs after receiving PrEP due to pre-existing HIV, no or inconsistent use of PrEP, PrEP failure, or discontinued PrEP use [19, 37]. The actionable indicators such as PrEP usage, the quantity of PrEP dispensed, the number of new PrEP users, and active vs inactive PrEP users allowed direct monitoring of effective use of the program. These indicators can help ensure a sufficient and uninterrupted supply of PrEP, forecast potential demand for PrEP and identify gaps in PrEP needs and access. Active syphilis infection is a potent risk factor for HIV incidence and consequently benefits from PrEP use [38]. However, studies have identified high syphilis incidence and some adverse drug reactions among PrEP users [19, 39]. The observed associations of PrEP use and syphilis risk in those studies were potentially mediated by their behavioural change after PrEP initiation [39] and having a history of syphilis infection. Thus, routine monitoring of syphilis incidence and adverse drug reactions could help understand PrEP discontinuation and interruptions and decide on actions to take for safety measures.

Even though the global awareness of the role of PrEP in preventing HIV infection is well-established, recent literature regarding the implementation, monitoring and evaluation of PrEP strategies is limited [19]. We proposed monitoring indicators that have clear definitions and are easy to interpret, thereby demonstrating the feasibility of standardizing indicators to monitor any PrEP program and facilitate communication across settings. However, adequate monitoring would require access to quality and reliable data. We calculated the proposed indicators from routinely generating linked de-identified population-level administrative live data maintained by the BC-CfE that facilitates the production of periodic PrEP monitoring reports. Real-time data further informs and underpins more rapid, relevant and timely decision-making and responses [40]. For example, the numerous indicators we calculated using our ongoing PrEP program data are likely affected by the COVID-19 pandemic due to interrupted PrEP service delivery. The sudden drop in the number of clients in our program from 2020 Q1 to 2020 Q2 explains the unexpected decrease in PrEP access due to the pandemic. The number of new clients enrolled by the family physician and nurse practitioner decreased during the pandemic, while those enrolled by the physician specialists increased. Pandemic-related government restrictions and regulations may have changed the client’s HA, enrolment, or PrEP dispensation. The indicators such as general characteristics, HIV risk factors of the clients, healthcare providers and their trends over time help us to identify such unexpected situations as well as clients and health authorities that need prioritization to improve the effective use of the program. Further studies on the effects of the pandemic on program delivery could be beneficial for more pertinent revision of the indicators.

We acknowledge and highlight the limitations. The proposed indicators were assessed against eight selection criteria; however, the involvement of subjective judgement of the Technical Monitoring Committee when making decisions may affect the reproducibility of the finalized indicators. Therefore, selecting a committee consisting of relevant expertise is critical. We did not consider race/ethnicity as a possible stratification factor for our monitoring indicators since this information was not asked in the PrEP Enrolment and Prescription Request Form from January 2018 to December 2020. However, collecting this information is imperative to assess any bias in accessing the PrEP program, and therefore, the PrEP Enrolment and Prescription Request Form was revised at the end of 2022, and it now collects information on race/ethnicity. Further, some data limitations are associated when calculating the indicators. We list the applicable limitations here to avoid defining indicators incorrectly, enable proper interpretation of the results for program monitoring, and enhance clarity when these indicators are used in other settings. The eligible people and the proportion of clients offered or declined PrEP could not capture and thus considered only those prescribed when creating indicators. There could be under-reporting of key qualifying HIV risk factors and other variables since they were based on provider-reported information, which we could not validate. Program discontinuations and PrEP usage types must be better captured due to information lag and provider underreporting. We address these issues by changing the reporting system to include discontinuation and usage information on PrEP refill prescription forms. Data on adverse drug reactions in our PrEP program is based on voluntary reports of clinicians, patients, and caregivers. Therefore, the data to populate these indicators are constantly being revised. Complete data collection would help calculate adverse drug reaction events as an indicator of monitoring the safe use of PrEP. The study findings should be interpreted with caution since reductions in the availability of non-essential medical services due to the COVID-19 pandemic since March 2020 could have impacted the available data for indicators such as reduced access to testing. Also, the proposed indicators only capture some PrEP-associated domains due to data unavailability. Therefore, the developed indicators ideally include potential barriers to access care, such as social support, stigma, ongoing risk behaviour, PrEP awareness, personal perception, and willingness to use PrEP, as indicated in other studies [41–43]. In addition, as put forward by the WHO, PrEP is expected to supplement existing harm-reduction initiatives [19]. Thus, information from outlets that give harm-reduction support to high-risk groups will likely improve indicators’ reliability and validity, providing impending changes in such attributes before they occur. Our online report that uses these indicators for monitoring the PrEP program describes data limitations in detail [11].

We explained the process of developing a comprehensive PrEP monitoring framework using evidence-based indicators, and we calculated the indicators using an administrative database with real-time information. Stratification of the indicators exposed variability in the proper use of PrEP among subpopulations, thus providing approaches to address subpopulation considerations in continuing the PrEP program and identifying potential HIV prevention choices. In addition, these indicators can be adapted to other settings to guide strategically targeted interventions and facilitate collaboration across jurisdictions.
